# Establishment and Comparison of Two Different Diagnostic Platforms for Detection of DENV1 NS1 Protein

**DOI:** 10.3390/ijms161126069

**Published:** 2015-11-24

**Authors:** Yin-Liang Tang, Chien-Yu Chiu, Chun-Yu Lin, Chung-Hao Huang, Yen-Hsu Chen, Raul V. Destura, Day-Yu Chao, Han-Chung Wu

**Affiliations:** 1 Graduate Institute of Life Sciences, National Defense Medical Center, Taipei 114, Taiwan; bigerliang@yahoo.com.tw; 2 Institute of Cellular and Organismic Biology, Academia Sinica, Taipei 115, Taiwan; chienyu412@gmail.com; 3 Division of Infectious Diseases, Department of Internal Medicine, Kaohsiung Medical University Hospital, Kaohsiung 807, Taiwan; infectionman@gmail.com (C.-Y.L.); locusthao@gmail.com (C.-H.H.); infchen@gmail.com (Y.-H.C.); 4 School of Medicine, Graduate Institute of Medicine, Sepsis Research Center, Kaohsiung Medical University, Kaohsiung 807, Taiwan; 5 Department of Biological Science and Technology, College of Biological Science and Technology, National Chiao Tung University, Hsinchu 300, Taiwan; 6 Institute of Molecular Biology and Biotechnology, National Institutes of Health and Philippine Genome Center, University of the Philippines, Manila 1000, Philippines; raul.destura@biotechmanila.upm.edu.ph; 7 Graduate Institute of Microbiology and Public Health, College of Veterinary Medicine, National Chung-Hsing University, Taichung 402, Taiwan; dychao@dragon.nchu.edu.tw

**Keywords:** dengue virus, nonstructural protein 1, monoclonal antibody, diagnosis

## Abstract

Dengue virus (DENV) infection is currently at pandemic levels, with populations in tropical and subtropical regions at greatest risk of infection. Early diagnosis and management remain the cornerstone for good clinical outcomes, thus efficient and accurate diagnostic technology in the early stage of the disease is urgently needed. Serotype-specific monoclonal antibodies (mAbs) against the DENV1 nonstructural protein 1 (NS1), DA12-4, DA13-2, and DA15-3, which were recently generated using the hybridoma technique, are suitable for use in diagnostic platforms. Immunofluorescence assay (IFA), enzyme-linked immunosorbent assay (ELISA) and Western blot analysis further confirmed the serotype specificity of these three monoclonal antibodies. The ELISA-based diagnostic platform was established using the combination of two highly sensitive mAbs (DA15-3 and DB20-6). The same combination was also used for the flow cytometry-based diagnostic platform. We report here the detection limits of flow cytometry-based and ELISA-based diagnostic platforms using these mAbs to be 0.1 and 1 ng/mL, respectively. The collected clinical patient serum samples were also assayed by these two serotyping diagnostic platforms. The sensitivity and specificity for detecting NS1 protein of DENV1 are 90% and 96%, respectively. The accuracy of our platform for testing clinical samples is more advanced than that of the two commercial NS1 diagnostic platforms. In conclusion, our platforms are suitable for the early detection of NS1 protein in DENV1 infected patients.

## 1. Introduction

Dengue virus is one of the most critical and life-threatening global viruses, with symptoms including mild dengue fever (DF) and severe dengue hemorrhage fever (DHF)/dengue shock syndrome (DSS) [1,2]. According to literature, an estimated 390 million people are infected worldwide. Among them, about 500,000 individuals will develop DHF, and possibly DSS. Importantly, the disease leads to 25,000 deaths annually [2,3]. Currently, there is no suitable anti-viral drug or vaccine for treating or preventing the dengue disease. Only supportive therapy is available for providing limited assistance [4].

Dengue virus is a member of the *Flaviviridae* family. There are several important members in this family including Japanese encephalitis virus (JEV), West Nile virus (WNV), yellow fever virus (YFV), St. Louis encephalitis (SLE), and Tick-borne encephalitis virus (TBEV), most of which cause severe diseases [5]. Dengue virus has four different serotypes (DENV1, DENV2, DENV3, and DENV4). Primary infection results in the acquisition of life-long adaptive immunity to the same serotype of dengue virus, but secondary infection by heterologous serotypes will induce antibody dependent enhancement (ADE), thereby causing severe diseases (DHF or DSS) [6].

Dengue virus particles exist as an icosahedron. The shell of the mature virus particle is composed of structural proteins, including envelope (E) protein, membrane (M) protein, and capsid (C) protein, while the lipid bilayer of the host cell also forms part of the viral structure. Inside the shell exists a single-stranded, positive-polarity RNA, approximately 11 kb in size. The entire viral genome consists of two major parts: structural genes (C, prM/M, E) and nonstructural genes (NS1, NS2A, NS2B, NS3, NS4A, NS4B, NS5) [7]. The structural proteins form a vehicle for the genetic material, while the majority of proteins translated from the nonstructural genes have mainly unknown roles.

The NS1 protein, a secreted glycoprotein released from infected cells, is used as a clinical indicator for dengue virus infection [8,9,10,11]. After infection, the dengue virus spreads into the bloodstream, and accumulation of the NS1 protein in the bloodstream is thus a good indicator of infection. Typical methods for confirming dengue infections include: isolation of virus during the viremia stage, extraction of viral RNA, detection of NS1 protein, and serological tests [12,13]. For virion detection or viral RNA extraction, DENV-infected subjects and medical workers need to be aware of the symptoms during the early onset of disease. However, the initial signs for dengue infection are always asymptomatic, ambiguous, or typical of other diseases. Although immunoglobulin (Ig) M and G are used as indices for most infectious diseases, IgM or IgG only appear three to five days after the onset of disease. The antibodies for detecting DENV serotypes by immunostaining may cross react with other members of *Flaviviridae* family. In contrast, NS1 can be detected as early as viral particles themselves, and exists in the bloodstream for about one week [10]. In addition, the specific antibody for NS1 eliminates cross reactivity concerns. Because of these advantages, NS1 detection is gaining more traction than conventional detection assays.

Various diagnostic platforms have been developed for the detection of NS1 from clinical samples. Most of these platforms utilize the sandwich format capture ELISA principle; such diagnostic tools include the following: Platelia dengue NS1 Ag test (Bio-Rad Laboratories, Marnes La Coquette, France) [14,15,16,17], Pan-E dengue early ELISA test (Panbio Diagnostics, Brisbane, Australia) [14], dot blot immunoassay (DBI) [18], DEN antigen detection kit (denKEY Blue kit; Globio Co., Beverly, MA, USA) [18], InBios DENV Detect NS1 ELISA kit [19], Dengue early ELISA (MyBioSource), and Dengue virus NS1 ELISA test kit (Euroimmun) [14,15,20,21,22]. Other platforms, such as the NS1 lateral flow rapid test (LFRT) [15] and paper-based ELISA [23], can detect NS1 in a short time with high sensitivity based on the lateral flow effect. However, the current commercial ELISA kits cannot distinguish the four different dengue serotypes and showed different sensitivities to NS1 protein from different serotypes. Studies have reported diagnostic platforms with serotyping capability, suggesting that these platforms could be used as an alternative for diagnosis of dengue infection [13,24].

Here, we describe the generation and characterization of novel mAbs, and the design of diagnostic platforms based on DENV1 serotype-specific mAbs. These platforms could be used to detect purified or naturally-occurring NS1 protein, and showed comparable detection limits and better performance than two other commercial kits. Our study has demonstrated the clinical benefit of our proprietary platform, which was able to detect NS1 proteins with high specificity, leading to more accurate diagnosis results of the dengue infected patients. Thus, our detection platform may be a viable alternative for early detection of dengue virus infection.

## 2. Results

### 2.1. Purification of DENV1 NS1 Protein

To obtain purified DENV1 NS1 protein, an anti-NS1 antibody generated by our laboratory previously [25], DB16-1, was immobilized on NHS-activated Sepharose 4 Fast Flow beads, which were loaded onto a column. Next, DENV1-infected C6/36 cell culture supernatant was applied to the column, and the eluents were analyzed by different methods (Figure S1).

### 2.2. Generation and Identification of mAbs against DENV1 NS1 Protein

**Figure 1:**
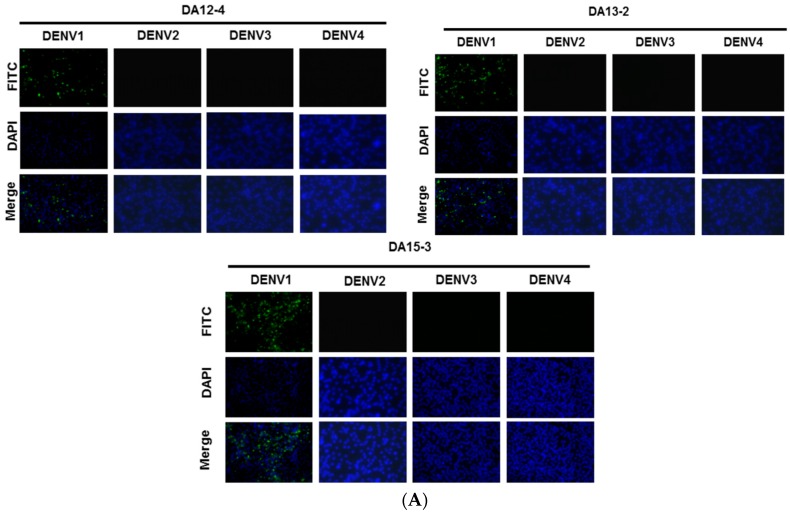

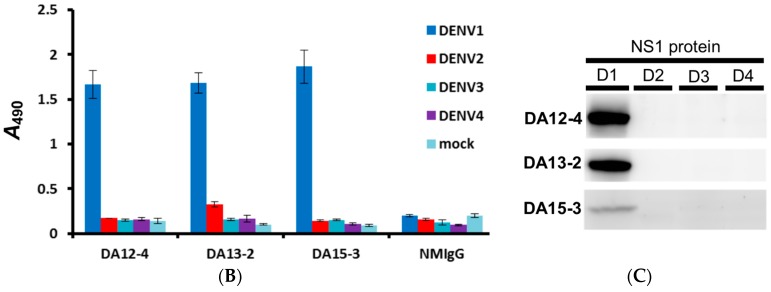
Identification of mAbs against NS1 protein by immunofluorescence assay, cellular ELISA, and Western blot analysis. (**A**) The indicated mAbs (DA12-4, DA13-2, and DA15-3) were individually incubated with BHK21 cells infected with DENV1 virus (strain Hawaii), DENV2 virus (strain 16681), DENV3 virus (strain H87) and DENV4 virus (strain H241). Magnification 400×. After secondary antibody and DAPI staining, the signals were detected by immunofluorescence microscopy; (**B**) C6/36 cells infected with the indicated serotype were incubated with the indicated mAbs for further analysis by cellular ELISA; (**C**) Lysates from C6/36 cells infected with the indicated serotypes of DENV were separated on a non-reducing gel, D1: DENV1-infected C6/36 cell lysate; D2: DENV2-infected C6/36 cell lysate; D3: DENV3-infected C6/36 cell lysate; D4: DENV4-infected C6/36 cell lysate. DENV1 NS1 protein was recognized by DA12-4, DA13-2, and DA15-3.

Purified DENV1 NS1 protein was used to immunize mice to generate mAbs. Three mAbs (DA12-4, DA13-2, and DA15-3) were characterized using IFA, ELISA, and Western blot analysis. The IFA results indicate that these mAbs can bind to DENV1-infected BHK21 cells (Figure 1A). The specificity of these mAbs was further examined by ELISA, and the results showed that these mAbs could specifically recognize DENV1 (Figure 1B). Western blot analysis was subsequently used to confirm that NS1 protein of DENV1 is the target of these mAbs (Figure 1C). In conclusion, all of these mAbs can specifically recognize the NS1 protein of DENV1, but not cross-react with NS1 proteins from other serotypes (Figure 1 and Table 1).

**Table 1:** Characterization of DENV1 mAbs by IFA, ELISA, WB.

mAbs	Isotype, Light chain	Specificity	IFA	ELISA				WB			
			D1	D1	D2	D3	D4	D1	D2	D3	D4
DA12-4	IgG2b, λ	NS1	+	+	−	−	−	+	−	−	−
DA13-2	IgG1, κ	NS1	+	+	−	−	−	+	−	−	−
DA15-3	IgG1, κ	NS1	+	+	−	−	−	+	−	−	−

mAbs, monoclonal antibodies; IFA, immunofluorescence assay; ELISA, enzyme-linked immunosorbent assay; WB, Western Blot analysis; D1, D2, D3, and D4, DENV1 to DENV4; Ig, immunoglobulin; NS1, nonstructural protein 1. (+) positive result to DENV, *A*_490_ (absorbance at 490 nm) > 0.2; (−) negative result to DENV, *A*_490_ < 0.2.

### 2.3. Establishment of an ELISA-Based Diagnostic Platform

**Figure 2:**
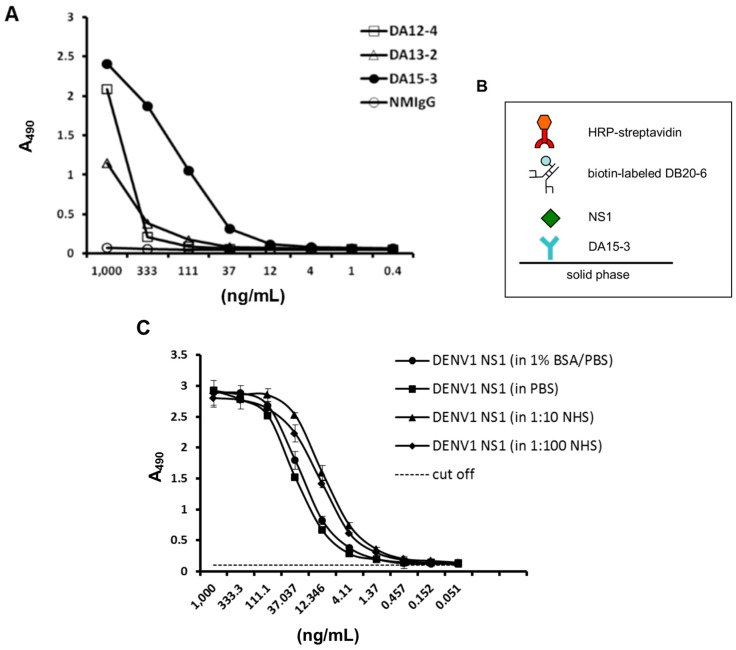
Development of an ELISA-based diagnostic platform for DENV1. (**A**) Direct ELISA was used to compare a panel of mAbs against serial dilutions of immunoaffinity-purified DENV1 NS1 protein. The ELISA plate was coated with a three-fold dilution of purified DENV1 NS1 protein. After washing, the coated NS1 protein was detected with the indicated mAb at a concentration of 1 μg/mL. Normal mouse IgG (NM-IgG) was used as the negative control; (**B**) Schematic describing the diagnostic platform; (**C**) Standard curve of DENV1 NS1 in different buffer systems. DENV1 NS1-specific mAb, DA15-3, was used to coat an ELISA plate at a concentration of 50 μg/mL. Immunoaffinity-purified DENV1 NS1 protein was diluted three-fold in different buffers and incubated with the capture mAb. NS1 protein was detected through the scheme shown in panel (**B**). Data points represent the mean ± standard deviation for three replicates. The dashed line represents the cut off value. NHS: normal human serum.

The ELISA-based diagnostic platform was constructed based on the concept of capture ELISA. Optimization of the detection limit of this platform required consideration of the affinity of these mAbs. The results from regular direct ELISA indicate that DA15-3 possessed the highest binding affinity of the mAbs, and was thus utilized as the capture antibody for serotyping purpose (Figure 2A). The detection antibody for use in this platform should be able to recognize NS1 from all four serotypes of dengue virus since the prospective serotyping diagnostic platform could differentiate the serotypes based on the four different capture mAbs. As such, a biotin-labeled, cross-reactive mAb, DB20-6 generated by our laboratory previously was used for this purpose. A model of the ELISA-based diagnostic platform is shown in Figure 2B.

To determine the detection limit of this platform, we examined its ability to detect serially-diluted, immunoaffinity-purified DENV1 NS1. According to the ELISA results, the detection limit is 1 ng/mL (Figure 2C). Next, we investigated the reliability of this platform. Since the composition of blood is complex, we examined the effects of diluting purified DENV1 NS1 protein in different buffer systems. The titration curves were similar using each buffer, and the sensitivity of detection was unaffected by different buffer (Figure 2C). Therefore, this platform is reliable at detecting DENV1 NS1 protein under different conditions.

### 2.4. Establishment of a Flow Cytometry-Based Diagnostic Platform

**Figure 3:**
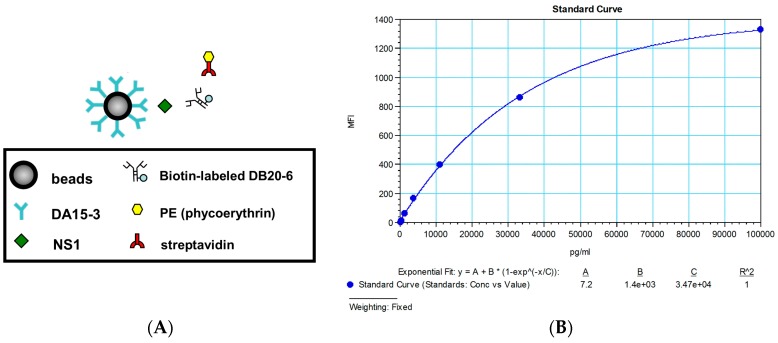
Development of a flow cytometry-based diagnostic platform for DENV1. (**A**) Schematic describing the diagnostic platform; (**B**) Standard curve of DENV1 NS1 for the flow cytometry-based diagnostic platform. MFI means the mean fluorescence intensity.

Flow cytometry was used to further strength the power of detection, by exploiting the high sensitivity of this technique. The basic principle of this model is depicted in Figure 3A. The DENV1 serotype-specific mAbs, DA15-3, was used as a capture antibody and coated on the surface of the beads. After the target protein (DENV1 NS1) is caught, biotin-labeled DB20-6 is detected and recognized using phycoerythrin (PE)-labeled streptavidin, and then analyzed by flow cytometry.

To establish the standard curve and explore the detection limit of this model, we serially diluted immunoaffinity-purified DENV1 NS1 protein, and incubated it with DA15-3-coated beads. Detection by flow cytometry produced a standard curve with a detection limit is 100 pg/mL, which is more sensitive than the ELISA-based platform (Figure 3B).

### 2.5. Detection of NS1 Protein in Sera from Patients Infected with DENV1

The importance of the NS1 protein in the diagnosis of dengue virus infection has been established in the literature [9,10,12]. The amount of NS1 protein is also related to autoimmunity and suggested a role in pathogenesis [25,26]. Thus we tried to detect the NS1 protein in the collected DENV1 patient blood samples using our platforms to see if these diagnostic platforms were useful in clinical applications.

**Figure 4:**
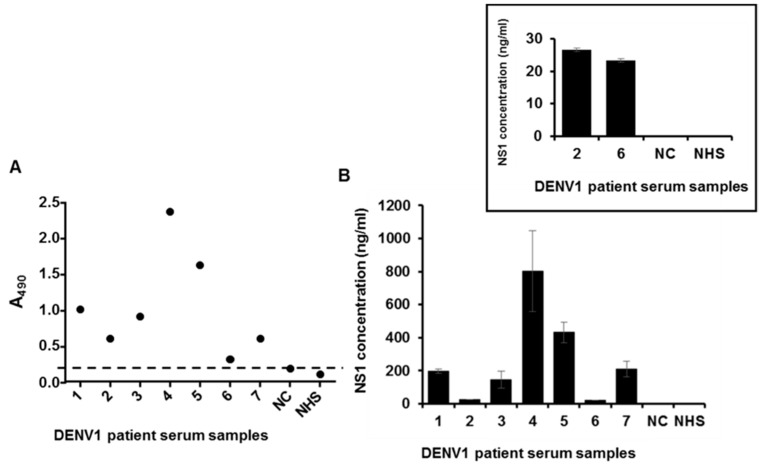

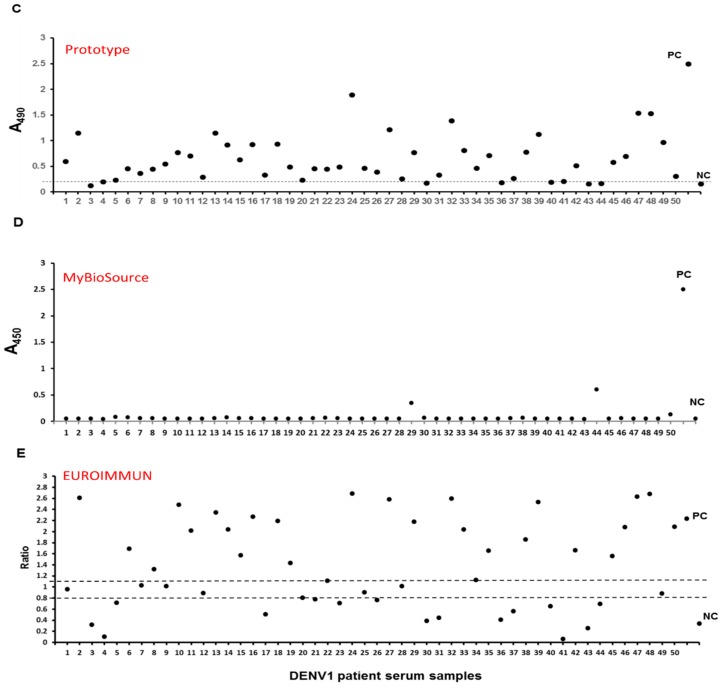
Detection of NS1 in sera from patients infected with DENV1. (**A**) Detection of NS1 in sera of patients infected with DENV1 using the ELISA-based diagnostic platform. NC represents RT-PCR negative control serum; NHS represents normal human serum. The dashed line represents the cut off value; (**B**) Detection of NS1 in the sera of patients infected with DENV1 using the flow cytometry-based diagnostic platform. NC represents RT-PCR negative control serum; NHS represents normal human serum; (**C**) Fifty serum samples were collected and analyzed by the ELISA-based diagnostic platform. Diluted normal human serum (1 to 100 in Thermo IgG elution buffer) with the purified DENV1 NS1 protein (1 µg) as the positive control (PC); diluted normal human serum (1 to 100 in Thermo IgG elution buffer) without the purified DENV1 NS1 protein as the negative control (NC). The dashed line represents the cut off value which was set at the average value of 50 NHS plus triple standard deviation; (**D**) Fifty serum samples were analyzes by the commercial kit (MyBioSource), PC, positive control; NC, negative control; (**E**) Fifty serum samples were analyzed by the commercial kit (EUROIMMUN). The y-axis is the ratio of each sample or controls (Ratio = extinction of the control or patient sample/extinction of calibrator 2). Ratio ≥ 1.1: positive; Ratio ≥ 0.8 to <1.1: borderline; Ratio < 0.8: negative. PC, positive control; NC, negative control. Above data were repeated by two to three independent experiments.

Seven serum samples of DENV1 patients were from the Philippines and the NS1 protein was detected by the ELISA-based diagnostic platform. The data showed that the platform is able to detect DENV1 NS1 in clinical samples (Figure 4A). The same panel of clinical samples was also detected by the flow cytometry-based diagnostic platform, with a pattern similar to that of the ELISA-based platform (Figure 4B).

In 2014, there was a serious outbreak of dengue virus type 1 in southern Taiwan, with over 15,000 confirmed cases, 18 times greater than that in the previous year (2013). We cooperated with the local medical center (Kaohsiung Medical University Hospital) and used the ELISA-based diagnostic platform to test serum samples from 50 patients confirmed to have been infected with dengue virus. After incorporating the test results from additional 50 serum samples obtained from healthy volunteers, the sensitivity and specificity are 90% and 96%, respectively (Figure 4C). Two commercial NS1 protein detection kits were purchased and compared with our platform based on these serum samples. The results suggested that our diagnostic platform displayed better performance (Figure 4D,E, Table 2). The platform accurately detected NS1 protein in serum samples of DENV1 patients, providing direct evidence for the diagnostic potential of our detection system.

**Table 2:** Sensitivity and specificity of three different NS1 ELISA platforms.

	DENV1 NS1 ELISA kit		
	Prototype platform	Mybiosource (Dengue early ELISA)	Euroimmun (Dengue virus NS1 ELISA)
Sensitivity	90% (45/50)	4% (2/50)	54% (27/50)
Specificity	96% (48/50)	N/A	N/A

N/A: not assayed.

**Figure 5:**
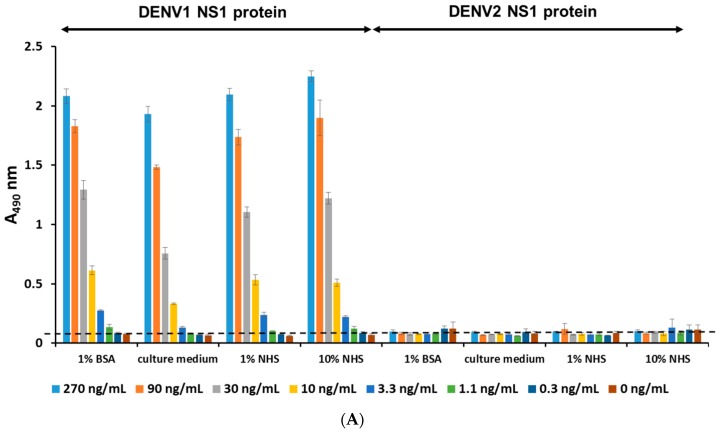

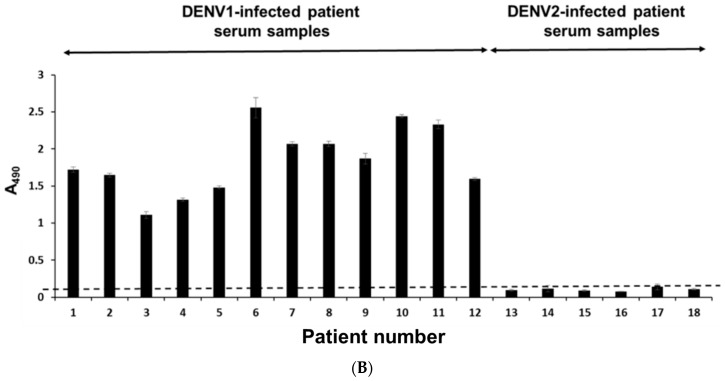
Serotyping specificity of DENV1 NS1 diagnostic platform. (**A**) DA15-3 was coated in the 96-well plate, serially-diluted immunoaffinity-purified DENV1 and DENV2 NS1 proteins were dissolved in different buffer systems and incubated with the coated mAb. After detection by biotin-DB20-6 and HRP-streptavidin, the signals were detected by spectrophotometer at 490 nm after the substrate processed by HRP; (**B**) The NS1 protein of serum samples from patients infected with DENV1 or DENV2 were detected using DENV1 NS1 diagnostic platforms. Dashed lines represented the cut off value.

### 2.6. Serotyping Specificity of the NS1 Diagnostic Platform

To confirm the serotyping specificity of our platform, we used it to detect NS1 protein in human serum. We found that our platform could detect the NS1 protein of DENV1 with high specificity, but not the NS1 protein of DENV2 (Figure 5A). We further demonstrated that our platform can detect the NS1 protein in DENV1-infected patient sera, but not in DENV2-infected patient sera (Figure 5B). Our results demonstrate that our platform can detect the NS1 protein of DENV1 with high specificity, without showing any cross-reactivity to the NS1 protein of DENV2.

## 3. Discussion

Dengue virus causes serious problems worldwide. Currently, there is no serotype-specific diagnostic reagent for the detection of viral antigens available commercially. In this study, we have developed a DENV1 serotype-specific diagnostic platform that can enable early detection of DENV1 infection. We have presented two different platforms for the detection of DENV1 NS1 protein. The ELISA format possessed a detection limit of 1 ng/mL, which is more sensitive than previously described platforms (4 ng/mL) [11]. Our second platform, based on flow cytometry, exhibited an even higher sensitivity, with a detection limit of 100 pg/mL. Other non-commercialized platforms in the literature utilized the similar principle and showed serotyping specificity [13,24]. However, our study focused on the improvement of the detection limits through our newly generated highly sensitive mAbs, which replaced IgM used in another study to enhance the capture ability [13] and simplified the operating procedures of the existing diagnostic platforms [24].

According to the epidemiology, DENV1 and DENV2 appeared more frequently than the other two serotypes (DENV3 and DENV4). Most early detection systems focus on NS1 protein, due to its early appearance and relative stability. We choose DENV1 to be our target and DA15-3 as capture antibody because this antibody has highest NS1 protein reactivity in ELISA (Figure 1B) and immunofluorescent assay (Figure 1A) without interfering with the binding of DB20-6 (Figure 2B). However, DA15-3 showed weaker response in Western Blot analysis, suggesting that DA15-3 might bind to the conformational epitope on the NS1 protein.

Flow cytometry has been applied to analyze bone marrow aspirates for routine staging of non-Hodgkin lymphoma (NHL) patients or immunophenotyping of leukemia [27,28]. Therefore, in this study, we leveraged the same flow cytometry technology platform to detect dengue viral antigen. Our design involved coating the capture mAb (DA15-3) to the surface of beads. The distribution of DA15-3 showed higher space availability than that coated on a planar solid phase, because of the steric hindrance. Due to the higher probability of DA15-3 capturing NS1 (as compared to other antibodies), the detection limit of this platform is higher than that of regular capture ELISA assays. In the future, if the same technology platform can be developed for the other three DENV serotypes, it will be possible to rapidly detect the specific serotype of the DENV infection with high sensitivity.

In this study, we analyzed seven clinical serum samples from patients infected with DENV1 in the Philippines and 50 clinical serum samples from patients infected with DENV1 during the outbreak in Taiwan in 2014. These blood samples were diluted at a factor of 1 to 100 and treated with acid solution to expose the epitope of target protein after dissociation of the immune complex [29,30,31]. Our results suggest that our platform has high sensitivity and accuracy. The lower readout values of certain samples are most likely due to the presence of a lower amount of NS1 protein, or the immune complex being relatively stable under acidic conditions. In this study, we also compared our NS1 diagnostic platform with two other commercial DENV NS1 detection kits (MyBioSource and EUROIMMUN). Based on the results, our platform demonstrated superior sensitivity for the early detection of DENV1 infection compared to the two commercially available kits; EUROIMMUN and MyBioSource (Figure 4D,E, Table 2). Furthermore, our platform could specifically detect NS1 from DENV1 (Figure 5A,B), which are lacking from the commercially available diagnostic kits.

## 4. Experimental Section

### 4.1. Cells and Viruses

BHK21 cells were cultured in Minimal Essential Medium (MEM, Gibco, Grand Island, NY, USA) with 10% fetal bovine serum (FBS, Gibco) and antibiotic-antimycotic (Gibco) at 37 °C with 5% CO_2_. *Aedes albopictus* C6/36 cells were cultured in a 1:1 ratio of Dulbecco’s modified Eagle’s medium (DMEM, Gibco) and Mitsuhashi and Maramorosch (MM) insect medium (Sigma-Aldrich, St. Louis, MO, USA) containing 10% FBS and antibiotic-antimycotic at 28 °C. Four serotypes of DENV (DENV1 Hawaii, DENV2 16681, DENV3 H87, DENV4 H241) were provided by Dr. Duane J. Gubler of the Centers for Disease Control and Prevention, Fort Collins, CO, USA.

### 4.2. Human Serum Samples

Anonymized human serum samples were kindly provided by Dr. Yen-Hsu Chen (Taiwan) and Dr. Raul V. Destura (Philippines). Control serum samples were provided by healthy donors. The study protocols were approved by the Kaohsiung Medical University Hospital Institutional Review Board (KMUHIRB-2011-04-02(I)) and University of the Philippines Institutional Review Board (NIH 2010-047). All clinical serum samples were confirmed to be positive by real time-PCR or BioRad NS1 rapid test.

### 4.3. Virus Infection

C6/36 cells were seeded in T75 flasks (1 × 10^7^ cells/flask). When the cell density approached 80%~90% confluence, the 10% FBS-containing culture media were replaced with 2% FBS-containing culture media, and virus (DENV1, strain Hawaii) at a multiplicity of infection (M.O.I.) of 0.1 was used to inoculate the culture. The C6/36 cells were then incubated at 28 °C for 5~7 days. Subsequently, culture media were centrifuged at 800× *g* for 5 min at 4 °C, and the supernatant was stored at −80 °C until use.

### 4.4. DENV Antigen Preparation

C6/36 cells were infected with one of four serotypes of DENV. Infected cells were lysed in lysis buffer (25 mM Tris-HCl, pH 7.4, 150 mM NaCl, and 1% Nonidet P-40) containing protease inhibitors (Roche, Mannheim, Germany). Cell debris was removed by centrifugation at 3000× *g* for 10 min at 4 °C, and the protein concentration of the supernatant was determined by using a UV spectrophotometer to measure absorbance at 280 nm.

### 4.5. Purification of DENV1 NS1 Protein

NHS-activated Sepharose 4 Fast Flow beads (GE Healthcare Cat# 17-0906-01, Uppsala, Sweden) were utilized to purify DENV1 NS1 protein. Briefly, the beads were packaged into the column and washed by ddH_2_O. Beads were activated using freshly-prepared and chilled HCl solution (1 mM), and then washed with PBS. The beads were then mixed with anti-NS1 mAb (DB16-1) and incubated at room temperature for 2 h. The mAb was removed, and the beads were washed with PBS; subsequently, the un-reacted sites on beads were blocked using 0.5 M Tris buffer (pH 8) at room temperature for 1 h. The beads were washed with three different buffers, in the following order: PBS, 0.1 M Tris (pH 9)/0.5 M NaCl, PBS, 0.1 M glycine (pH 2.3)/1 M NaCl. The wash cycle was repeated three times, and the beads were then equilibrated with PBS. Next, harvested DENV1-infected C6/36 cell supernatant was applied to the column, which was then washed with PBS. Captured DENV1 NS1 protein was eluted with 0.1 M glycine (pH 2.3), and fractions were harvested. Finally, each fraction was neutralized with 1 M Tris (pH 9), and protein concentration was determined based on absorbance at 280 nm. Purification of DENV2 NS1 protein also follows the same procedures.

### 4.6. Generation of mAbs against DENV1 NS1 Protein

The protocol for generating mAbs against DENV1 NS1 protein was based on the procedures described in a previous study [25]. Briefly, female BALB/c mice were immunized intraperitoneally with purified DENV1 NS1 protein at 3-week intervals for a total of four immunizations. Lymphocytes were harvested from the spleen of the immunized mouse on day 4 after the final boost, and then the harvested lymphocytes were subsequently fused with NSI/1-Ag4-1 myeloma cells using 50% polyethylene glycol (PEG 1500; Roche, Indianapolis, IN, USA) by adding 50% PEG drop by drop and mixed the cell mixture at the fusion step. The fused cells were resuspended in DMEM containing 20% FBS, hypoxanthine-aminopterin-thymidine, and hybridoma cloning factor (ICN Biomedicals, Aurora, OH, USA). All animal experiments were performed in accordance with the guidelines of the National Laboratory Animal Center. The protocol was approved by the Committee on the Ethics of Animal Experiments of Academia Sinica. Cultured hybridoma supernatant was incubated with DENV1-infected C6/36 cells to confirm the specificity of antibodies, as described under “Cellular ELISA”. Hybridoma cell lines were grown in DMEM with 10% FBS. Ascites were generated in pristine-primed BALB/c mice, and mAbs were purified using protein G-Sepharose 4 Fast Flow gel.

### 4.7. Immunofluorescence Assay (IFA)

BHK21 cells were seeded on coverslips (Deckglaser Cover Glasses, 12 mm in diameter) in a 24-well plate, with each well containing 1 × 10^5^ cells, and then the BHK21 cell-seeded plate was incubated at 37 °C overnight. Cells were infected with DENV1 virus (strain Hawaii) at an M.O.I. of 0.5; the plates were subsequently incubated at 37 °C for 2 h, with shaking once every 30 min. After adding fresh 2% FBS-containing medium to each well, the plates were cultured at 37 °C for 2 days. The cells were washed twice with PBS after the infection period, and were then fixed and permeabilized using methanol/acetone (1:1). The plates were then incubated at −20 °C for 20 min, and methanol/acetone solution were subsequently removed using PBS. The cells were then incubated in blocking buffer (1% BSA/PBS) at room temperature for 30 min. Each mAb (DA12-4, DA13-2, or DA15-3) was separately diluted in blocking buffer (5 μg/mL), and the dilutions were incubated with infected cells at room temperature for 1 h. Finally, cells were washed and mounted on glass slides, covered with coverslips, and fixed using polish oil. A Zeiss microscope imaging system was used to visualize the cells.

### 4.8. Cellular ELISA (Enzyme-Linked Immunosorbent Assay)

After seeding C6/36 cells on 96-well plates (2 × 10^4^ cells/well), the plates were subsequently incubated at 37 °C overnight. After infecting the cells with DENV (one serotype of types 1 to 4) at an M.O.I. of 0.1, the cells were further incubated at 28 °C for 5 to 7 days. The cells were washed twice with PBS, fixed and permeabilized with methanol/acetone (1:1), and incubated at −20 °C for 20 min following the infection period. Next, the cells were washed three times with PBS, blocked with 5% skimmed milk, and incubated at 4 °C overnight. The cells were given a final wash with PBS prior to ELISA. After adding each individual mAb (DA12-4, DA13-2, DA15-3, or normal mouse IgG (NMIgG)) to each well (*i.e.*, each well containing only a single mAb), the plates were incubated at room temperature for 1 h. The cells were then washed three times with PBS. After adding the secondary antibody, HRP-conjugated goat anti-mouse immunoglobulin G (Jackson ImmunoResearch Laboratories), to the wells (1:2000 dilution), the plates were incubated at room temperature for a further 1 h. Cells were washed four times with PBS containing 0.1% Tween 20. Signals were developed using the peroxidase substrate, *o*-phenylenediamine dihydrochloride (OPD; Sigma-Aldrich, St. Louis, MO, USA). HCl at a concentration of 3 N was used to stop the reaction, and the plates were read using the Spectra Max M5 (Molecular Devices, Sunnyvale, CA, USA) microplate reader at an absorbance of 490 nm.

### 4.9. Western Blot Analysis

The lysates of C6/36 cells infected with DENV (serotypes 1 to 4; 5 µg/sample) were separated on a sodium dodecyl sulfate (SDS)-polyacrylamide gel (10%). Next, the proteins in the gel were separated and transferred onto nitrocellulose (NC) paper. The NC paper was blocked with 5% skimmed milk, and then different mAbs (DA12-4, DA13-2, or DA15-3) were hybridized to the NC paper. The bound mAbs were recognized by horseradish peroxidase (HRP)-conjugated goat against mouse immunoglobulin G (IgG), and signals were developed using enhanced chemiluminescence substrate (Millipore, Billerica, MA). Signals were recorded using a BioSpectra 600 Imaging System (UVP, Upland, CA, USA).

### 4.10. Direct ELISA

Serially-diluted purified DENV1 NS1 protein in coating buffer (0.1 M NaHCO_3_) was used to coat 96-well plates (50 μL/well) at room temperature for 2 h. The plates were blocked with 1% BSA/PBS at 4 °C overnight. After washing with PBS, NS1 was incubated with a mAb (DA12-4, DA13-2, or DA15-3), which was subsequently detected using HRP-conjugated goat against mouse IgG. Signals were developed using the peroxidase substrate, *O*-phenylenediamine dihydrochloride (OPD; Sigma-Aldrich, St. Louis, MO, USA), and read using a Spectra Max M5 (Molecular Devices, Sunnyvale, CA, USA) microplate reader at an absorbance of 490 nm.

### 4.11. Biotinylation of mAb

Biotinylation of DB20-6 was performed according to the instructions provided with EZ-Link NHS-Biotin Reagents (Thermo scientific, Rockford, IL, USA). Briefly, the biotin powder was dissolved in dimethyl sulfoxide (DMSO) solution to a final concentration of 10 mM, and then the appropriate volume (calculated using the formula provided by the manufacturer) was added to 1 mg DB20-6 dissolved in 200 μL of PBS. The mixture was incubated on ice for two hours or at room temperature for 30 min. The mixture was resuspended in PBS following Amicon (Millipore, Cork, Ireland) centrifugation, and protein concentration was measured using a UV spectrophotometer (Thermo Scientific, Rockford, IL, USA) as described above.

### 4.12. Establishment of NS1 Standard Curves

Capture antibody (DA15-3; 50 μg/mL) was used to coat a 96-well plate, which was subsequently incubated at 4 °C overnight. After incubation, the plate was washed with PBS and blocked with 1% BSA/PBS at room temperature for 2 h. Immunoaffinity-purified DENV1 NS1 protein was serially diluted in different buffer systems: PBS, 1% BSA/PBS, 1% normal human serum (NHS), or 10% NHS. After removing the blocking buffer by washing three times with PBS, serially-diluted NS1 protein was added to the wells and incubated at room temperature for 1 h. The plates were then washed three more times with PBS, followed by the addition of biotin-labeled DB20-6 (2 μg/mL) to the wells (50 μL/well). The plates were subsequently incubated at room temperature for 1 h. After incubation, the plates were washed three further times with PBS; HRP-conjugated streptavidin (1:1000) was then added to the wells. The plates were incubated at room temperature for 1 h, followed by four washes with PBST_0.1_ (PBS containing 0.1% Tween20). The signals were developed with the peroxidase substrate, *O*-phenylenediamine dihydrochloride (OPD; Sigma-Aldrich). The reaction was stopped by the addition of 3 N hydrochloric acid, and the plates were read using the Spectra Max M5 (Molecular Devices) microplate reader at an absorbance of 490 nm.

### 4.13. Detection of NS1 Protein in Clinical Samples

An ELISA plate was coated with 50 μg/mL of DA15-3 in 0.1 M sodium bicarbonate buffer (NaHCO_3_, pH 8.6) at room temperature for 2 h. After washing with PBS, the plate was blocked with blocking buffer (1% BSA in PBS) at 4 °C overnight. Clinical samples were diluted with Thermo IgG elution buffer (pH 2.8, Prod # 21009) for 20 min, and then neutralized with 1 M Tris (pH 9.0) at a final ratio of 1 to 100. Diluted normal human serum (1 to 100 in Thermo IgG elution buffer, Thermo Scientific, Rockford, IL, USA) with purified DENV1 NS1 protein (1 µg) as the positive control; diluted normal human serum (1 to 100 in Thermo IgG elution buffer) without the purified DENV1 NS1 protein as the negative control. The plate was washed with PBS, and then incubated with diluted clinical samples (50 μL/well) at room temperature for 1 h. Following another wash with PBS, the plate was incubated with biotin-labeled DB20-6 (2 μg/mL) at room temperature for 1 h. The plate was then washed with PBS, and hybridized with HRP-conjugated streptavidin (1:1000) at room temperature for 1 h. After a further four washes with PBST_0.1_, signals were developed by the addition of the peroxidase substrate, *O*-phenylenediamine dihydrochloride (OPD; Sigma-Aldrich). The reaction was stopped by the addition of 3 N HCl, and the plates were read using a Spectra Max M5 (Molecular Devices) microplate reader at an absorbance of 490 nm. The definition of cut-off value for the prototype diagnostic platform is the average readouts of 50 normal human serum samples plus three times of standard derivation.

### 4.14. Flow Cytometry Analysis

DA15-3, biotin-DB20-6, and purified DENV1 NS1 protein were used to construct a platform by YSL Bioprocess Development Co. (Pasadena, CA, USA). The experimental procedures were performed in accordance with the user manual. The capture beads (45 μL) were mixed with standard reference (45 μL) or samples (45 μL) for 60 min at room temperature and retrieved. The 100 μL wash buffer was then introduced to wash the recovered beads three times. This new set of beads was subsequently coated with the biotinylated antibody for 30 min at room temperature. This mixture was washed three times again with 100 μL wash buffer before 25 μL of streptavidin-PE solution was added for 20 min at room temperature. The beads were subsequently measured using flow cytometry.

### 4.15. Detection of NS1 Protein in Clinical Samples by Commercial Kits

Two commercial kits, dengue virus NS1 ELISA (EUROIMMUN, Luebeck, Germany) and dengue early ELISA (MyBioSource, Vancouver, BC, Canada) were purchased for comparison. All experimental procedures followed the test instructions of the kits.

## 5. Conclusions

Here, we report the generation of new mAbs and platforms to provide novel means of early detection of DENV1 infection. There remains an urgent need for the development of highly sensitive and serotype-specific diagnostic platforms that can provide early detection of other serotypes of dengue infections. The development of these high sensitivity platforms for early detection of dengue infection will benefit the study of epidemiology and the treatment of dengue disease.

## Supplementary Materials

Supplementary materials can be found at http://www.mdpi.com/1422-0067/16/11/26069/s1.
