# Supplementary material for: Establishment and Comparison of Two Different Diagnostic Platforms for Detection of DENV1 NS1 Protein
Source: Int J Mol Sci. 2015 Nov 24;16(11):27850–64. doi: 10.3390/ijms161126069 (PMC4661927; doi:10.3390/ijms161126069)
Supplement: Supplementary file 1 [file ijms-16-26069-s001.pdf]

Supplementary Materials: Establishment and Comparison of Two Different Diagnostic Platforms for Detection of DENV1 NS1 Protein

Yin-Liang Tang, Chien-Yu Chiu, Chun-Yu Lin, Chung-Hao Huang, Yen-Hsu Chen, Raul V. Destura, Day-Yu Chao and Han-Chung Wu

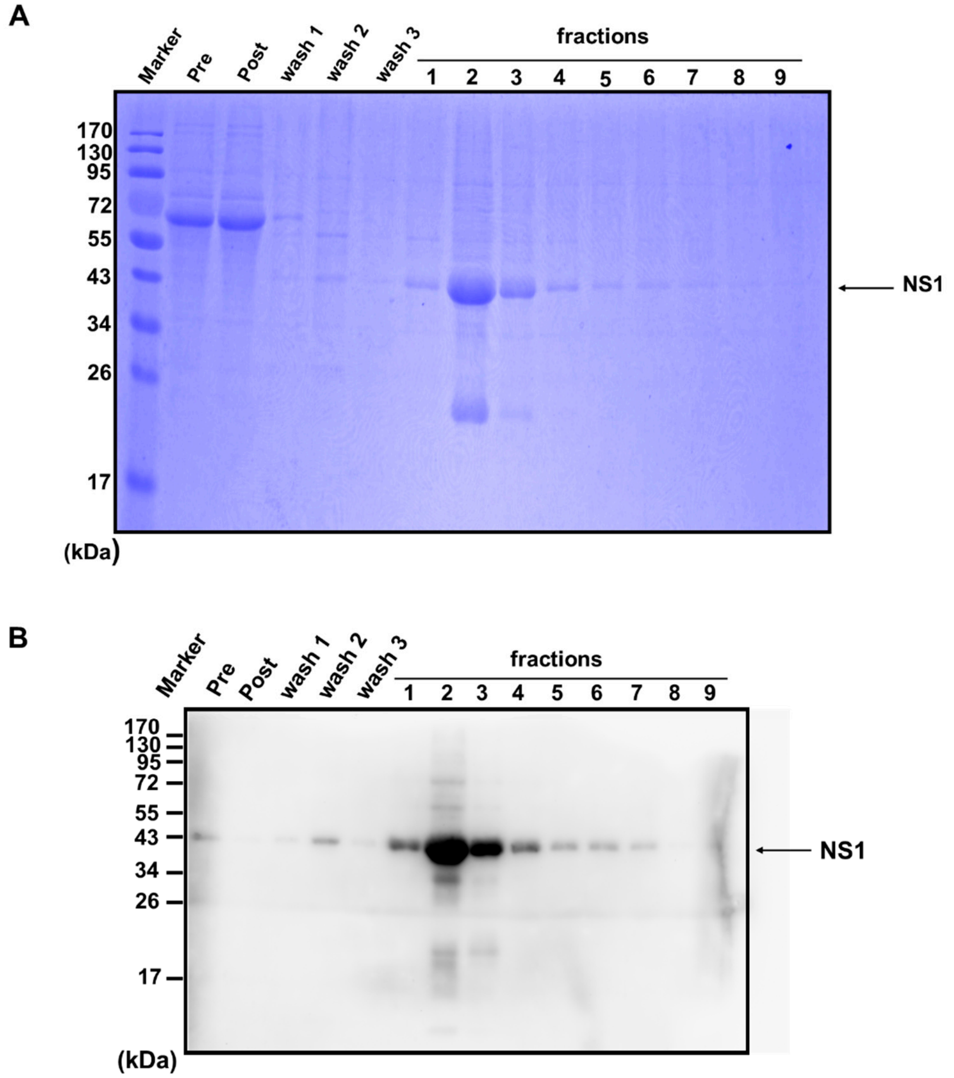

Figure S1. Purification of DENV1 NS1 protein from virus-infected C6/36 cell culture supernatant. Cross-reactive mAb DB16-1 was incubated with NHS-activated Sepharose 4 Fast Flow beads in a column. After blocking and washing, virus-infected C6/36 cell culture supernatant was applied to the column. NS1 was eluted and collected, as described in the Methods and Materials. The eluted proteins were further analyzed by Coomassie blue staining (**A**) and Western blot analysis (**B**).
